# Examining the Contributions of Parents’ Daily Hassles and Parenting Approaches to Children’s Behavior Problems during the COVID-19 Pandemic

**DOI:** 10.3390/children10020312

**Published:** 2023-02-07

**Authors:** Ibrahim H. Acar, Sevval Nur Sezer, İlayda Uculas, Fatma Ozge Unsal

**Affiliations:** 1 Department of Psychology, Faculty of Social Sciences, Çekmeköy Campus, Ozyeğin University, 34794 Istanbul, Turkey; 2 Department of Early Childhood Education, Faculty of Education, Göztepe Campus, Marmara University, 34722 Istanbul, Turkey

**Keywords:** parenting daily hassles, the COVID-19 pandemic, parenting approaches, behavior problems

## Abstract

The present study was designed to examine the direct and indirect contributions of parenting daily hassles and approaches to children’s externalizing and internalizing behavior problems during the COVID-19 pandemic. The sample for this study was 338 preschool children (53.6% girls, *M_age_* = 56.33 months, *SD* = 15.14) and their parents in Turkey. Parents reported their daily hassles, parenting approaches, and children’s behavior problems. Findings from the structural equation model showed that higher levels of parenting daily hassles predicted higher levels of externalizing and internalizing behavior problems. In addition, we found an indirect effect of daily hassles on children’s internalizing behaviors via positive parenting. Further, there was an indirect path from parenting daily hassles to children’s externalizing behaviors through the negative parenting approach. Results are discussed in the context of the COVID-19 pandemic.

## 1. Introduction

Unprecedented times and contexts, such as the COVID-19 pandemic, procreate adverse effects for specific groups, such as parents with young children because of their fragility, exacerbated burden in childcaring and sustaining within-family functioning [1,2]. Further, more than 1.6 billion children were out of school because of lockdown policies [3], which placed an additional burden on parents with young children. As a natural consequence of home confinement and within-family dysfunctions (e.g., juggling between childcare and work, dealing with children’s needs) due to the COVID-19 pandemic, children’s behavior problems have increased [4,5]. From this perspective, we aimed to investigate the daily hassles of parents related to parenting practices within the family and how this within-family functioning undermined children’s behavior problems.

Behavior problems in children can be grouped into internalizing (e.g., anxiety and depression) and externalizing behaviors (e.g., aggression and hyperactivity) [6]. As a consequence of the COVID-19 pandemic, children have experienced a lack of social interactions and movement restrictions, which have adversely affected their development [3]. For example, Jiao et al. [7] found that children felt insecure, frightened, and lonely during the pandemic, and also experienced sleep disturbances, nightmares, eating problems, agitation, inattention, and separation anxiety. Overall, young children have mainly been affected by the COVID-19 pandemic, as developmentally making sense of the situation could be more challenging for them [8,9,10].

As children experience the pandemic in a family context, within-family functioning reflecting parenting approaches and daily routines naturally affect children’s behavioral outcomes [2]. The change, stress, restrictions, and financial problems that came with the pandemic have increased the burden on parents and overall within-family interactions [1,11]. Because children require support from their parents in daily functioning to a large extent in the preschool period, parents with the burdens of the pandemic may lack support for their children, which could lead children to display behavior problems [1,2]. Considering the importance of parenting approaches and parenting stress within the family context on the development of children’s behavior problems, it is essential to examine the impacts of parents’ daily hassles and parenting approaches on children’s behavior problems during the COVID-19 pandemic.

### 1.1. Parents’ Socialization and Children’s Behavior Problems

The family context is the first social environment for children where they begin socializing [12]. Child-rearing approaches as part of the family social context play a critical role in the socialization of children, leading them to display culturally accepted behaviors. While positive child-rearing practices may contribute positively to the socialization of children, negative child-rearing approaches may prevent children’s socialization and pave the way for behavioral problems for children [13]. Parents utilize positive child-rearing practices such as inductive reasoning and warmth and negative practices such as punishment and obedience-demanding [14]. Practicing negative child-rearing approaches (i.e., punishment and obedience-demanding) may increase children’s risk of developing behavioral problems. For example, using physical punishment as a discipline and demanding obedience (e.g., expecting compliance without explanation) by parents has been positively linked to child behavior problems such as aggression [15]. On the other hand, using inductive reasoning (e.g., providing explanations to children) and warmth (e.g., showing acceptance and sensitivity to a child’s needs) has been linked to a decrease in children’s behavior problems [15]. Further, children whose interests and needs were not considered expressed anger by crying, stomping, and holding their breath to get what they wanted, reflecting behavior problems [15,16]. Overall, using overprotection and strict discipline as child-rearing approaches is associated with having children with internalizing and externalizing behavioral problems such as introversion, withdrawal, anxiety, and self-harm; on the other hand, adopting warmth and a positive parent–child relationship as child-rearing approaches was associated positively with the social development of children [16].

Grounded on the findings of previous studies, we can speculate that the child-rearing approaches adopted by the parents are effective in the development of behavioral problems of the children. However, the nature of the child-rearing approaches may have changed due to the adverse effects of the COVID-19 pandemic [17,18]. Confinement of parents may have placed an additional burden on parents with young children, leading them to display negative parenting practices [1].

### 1.2. Roles of Parenting Daily Hassles on Children’s Behavior Problems

Parents with young children have juggled multiple tasks during the pandemic, mainly dealing with daily routines. Hassles in daily functioning may have undermined parental well-being and the quality of parent–child interactions [19]. Daily parenting hassles refer to experiences of common day-to-day burdens that emerge from caring for children [20]. In detail, daily parenting hassles could be both the burdens that parents experience in parenting-related chores (e.g., running errands) and their children’s behaviors (e.g., picky eater) [19,21]. For example, the major daily hassles could be arranging bedtime routines, meal-time difficulties, cleaning toys, or sibling arguments, as well as other minor stressors in day-to-day living. Daily parenting hassles could undermine children’s internalizing and externalizing behavior problems. Results from previous studies showed that daily parenting hassles contributed to children’s behavior problems concurrently and longitudinally [22,23,24].

The COVID-19 pandemic may have increased daily hassles for parents, particularly parents with young children [25]. Knowing that daily hassles are critical underlying predictors of child-rearing practices and children’s well-being, it is important to examine their role in child behavior problems during the COVID-19 pandemic.

### 1.3. Link from Daily Hassles to Children’s Behavior Problems through Parenting Approaches

Understanding the needs of the children and responding to them appropriately is very important in terms of the psychological well-being of children and the quality of the bond they establish with their parents. The nature of parenting approaches could provide a socialization context for children to develop both positive and negative social outcomes [26,27]. Evidence is clear that positive parenting approaches, such as parental warmth, can make children feel secure and help children to display fewer behavior problems [28,29]. On the other hand, negative parenting approaches are often accompanied by punishment and obedience-demanding, which can increase children’s behavior problems [15,23].

Daily hassles in parenting may deplete the resources of parents and undermine their regulatory capacity, which may lead them to display negative parenting approaches [20,30]. In detail, daily hassles emerging from parenting roles and demands, affecting the psychological well-being of parents, may be associated with disruptive and less optimal parenting approaches, which in turn lead their children to display behavior problems [23]. For example, parents with high levels of hassles showed negative parenting approaches (e.g., less tolerance towards their children, over-controlling), which were naturally found to be associated with internalizing and externalizing behavior problems in children (e.g., anxiety, depression, withdrawal, hyperactivity, and aggression [24,31]. Furthermore, a study with Turkish families showed an indirect effect of daily parenting hassles on children’s social and aggressive behaviors through parenting approaches [23]. More specifically, parents with higher levels of daily hassles tended to display more physical punishment towards their children, which in turn predicted greater aggressive behavior in children. Everything considered, these findings highlight the social-contextual notion that children’s behavior problems can be influenced by parenting context [32].

#### Study Purpose and Hypotheses

The pandemic may have brought additional hassles in parents’ daily functioning, which may have undermined their capability to attune children’s needs, which could lead their children to display more behavioral problems. To our knowledge, there has been no research examining the roles of daily hassles and parenting approaches on children’s behavior problems during the COVID-19. Therefore, the purpose of the current study was to investigate the contributions of association parenting daily hassles parenting approaches to child behavior problems during the COVID-19 pandemic. We first hypothesized that daily hassles would be positively associated with children’s behavior problems. Second, we hypothesized that daily hassles would be positively associated with negative parenting approaches and inversely associated with positive parenting approaches. Third, we hypothesized that positive parenting approaches would be negatively and that negative parenting approaches would be positively associated with children’s behavior problems. Fourth, we hypothesized that daily hassles would undermine positive parenting and exacerbate negative parenting, leading children to display higher levels of behavior problems.

## 2. Materials and Methods

### 2.1. Participants

The sample for the current study was 338 Turkish preschool children (53.6% girls, *M_age_* = 56.33 months, *SD* = 15.14). Only 11 of the participants were fathers; the remaining were mothers. Mothers’ age ranged from 24 to 47 years (*M* = 35.01, *SD* = 4.14) and fathers’ age ranged from 28 to 50 (*M* = 37.64, *SD* = 4.46). A total of 43.5% of mothers were employed, while 98.8% of fathers were employed during the pandemic. Family income was reported in blocks of TRY 1000 (Turkish liras) (~USD 117) and ranged from TRY 1000–2000 to more than TRY 7000/month, with a mode of TRY 7000 and higher. Employed mothers reported higher levels of parenting daily chores (*M* = 2.04, *SD* = 0.44; *M* = 1.91, *SD* = 0.45), *t*(336) = 2.67, *p* < 0.01) and behaviors (*M* = 2.13, *SD* = 0.56; *M* = 1.99, *SD* = 0.54, respectively, *t*(336) = 2.34, *p* < 0.01) compared to unemployed parents. The socioeconomic (SES) variable was created by averaging standardized (z-transformations) family income and education levels.

### 2.2. Materials

#### 2.2.1. Behavior Problems

Parents reported on the Child Behavior Checklist (CBCL) [6]. The CBCL has 100 items and is rated on a 3-point Likert-like scale (0 = Not True, 1 = Somewhat or Sometimes True, 2 = Very True or Often True). The CBCL has been validated and used with Turkish children [33,34]. The CBCL consists of internalizing behaviors (e.g., “unhappy, sad, or depressed”) and externalizing behaviors (e.g., “destroys things belonging to his/her family or other children”) subscales. Cronbach’s alpha was α = 0.87 for externalizing behaviors and α = 0.91 for internalizing behaviors. We summed items to create subscales where higher scores indicated higher levels of the construct.

#### 2.2.2. Parenting Approaches

Parents reported on the Child-Rearing Questionnaire (CRQ) [14]. The CRQ is a 30-item scale rated on a 5-point Likert-type scale (1 = Never and 5 = Always). The CRQ has been validated and used with Turkish parents [23,26,35]. The CRQ has four subscales measuring warmth (e.g., “*My child and I have warm, intimate times together*”), inductive reasoning (e.g., “*I try to explain to my child why certain things are necessary*”), punishment (e.g., “*I use physical punishment, e.g., smacking, for very bad behavior*”), and obedience-demanding (e.g., “*I expect my child to do what he/she is told to do, without stopping to argue about it*”). Internal consistency scores (Cronbach’s alpha) for inductive reasoning, punishment, obedience-demanding, and warmth were 0.80, 0.68, 0.79, and 0.87, respectively. We created positive (inductive reasoning and warmth) and negative (punishment and obedience-demanding) parenting latent constructs. See the Section 3 for the measurement model and factor loading values.

#### 2.2.3. Parenting Daily Hassles

Parents reported on the Parenting Daily Hassles Scale (PDHS) [19]. The PDHS is a 20-item scale rated on a 4-point Likert-type scale (1 = Rarely and 4 = Constantly). The PDHS has been validated and used with Turkish parents [23,36]. Taylor [21] reexamined the structure of the 20-item PDHS and recommended using two subscales: behaviors (e.g., “*Need to keep a constant eye on what kids are doing*”) and parenting chores (e.g., “*Kids get dirty and need to have clothes changed*”). Correspondingly, we used these two subscales in the current study to assess parents’ daily hassles. Internal consistency scores (Cronbach’s alpha) for behaviors and parenting chores were 0.82 and 0.80, respectively. We averaged items to create subscales where higher scores indicated higher levels of the construct.

#### 2.2.4. Fear of COVID-19

We used the Fear of COVID-19 Scale (FCV-19S). [36]. The FCV-19S is a 7-item scale rated on a 5-point Likert-type scale (1 = Strongly disagree and 5 = Strongly agree) (e.g., “*It makes me uncomfortable to think about coronavirus-19*”). The FCV-19S has been validated and used with Turkish participants [37,38]. The internal consistency score (Cronbach’s alpha) for the scale was 0.85. We averaged items to create a composite score where higher scores indicated higher levels of the construct. We used this construct as a covariate in our analyses.

### 2.3. Procedure

Following the University Human Subjects Ethics Committee’s approval on 22 March 2021(code: 2021/06/03), we created two data collection methods. First, we distributed consent forms and questionnaires to parents through child-care centers and parenting groups. Parents who voluntarily wanted to participate in this study returned their consent forms and completed questionnaires. Second, we created an online survey through Qualtrics. This step helped us reach out to more parents during the COVID-19 pandemic. In online data collection, aligned with paper-pencil data collection procedures, participants signed their consent before completing the questionnaires. We utilized the chain-referral sampling technique in both methods, where primarily contacted participants enabled us to find additional participants.

### 2.4. Data Analysis

We tested normality assumptions by using the criteria of +3 and −3 for skewness and +10 and −10 for kurtosis [39]. Our variables were within the acceptable range; therefore, no transformation was employed. See Table 1. Multivariate analyses were conducted in *Mplus 8.4* [40], using structural equation modeling (SEM) with maximum likelihood estimation. We followed the two-step model-building approach [40]. First, we tested the measurement model where we created latent factors of parenting daily hassles and parenting approaches (positive and negative). Once the measurement model fit the data, we tested the hypothesized structural model to examine whether there is an indirect effect of parenting daily hassles on children’s internalizing and externalizing behaviors through parenting approaches. We utilized top-down model building, where we included all possible covariates, including age, child sex, SES, and fear of COVID-19 in the model. We then removed the nonsignificant ones by considering the model fit improvement. We tested the significance of the indirect effects by using the bootstrapping technique (2000 resampling) with 95% confidence intervals [41]. We utilized a 95% bias-corrected bootstrap method, providing parsimonious results [40,41]. In line with Kline’s [40] recommendations, comparative fit index (CFI) and Tucker–Lewis index (TLI) values greater than 0.90, root mean square error of approximation (RMSEA) values of 0.08 or below, and standardized root means square residual (SRMR) values of 0.08 or below were employed to indicate a good fit [41,42]. Finally, we collected the data from the same respondents by using self-reported surveys at one point in time, and some measures could involve similar notional items, which may have created a common method bias [43] We used Harman’s single-factor test to see whether common method variance was present or not. The post hoc Harman’s single-factor test was used to check if a single factor is accountable for variance in the data [44]. The results from Harman’s single-factor test showed that only 18.23% of the variance was captured with the first unrotated factor (<50%), indicating that common method variance was not an issue in this study [44].

## 3. Results

Initially, we analyzed the bivariate correlations (Pearson) among study variables. Results showed that externalizing and internalizing behaviors were correlated with inductive reasoning, warmth, obedience, and punishment as part of the parenting approaches as well as parenting daily hassles. See Table 1 for complete correlation results.

### 3.1. Measurement Model

We tested the measurement model in *Mplus 8.4* [40] by creating latent variables of positive parenting, which consisted of warmth and inductive reasoning subscales; negative parenting, which consisted of obedience and punishment subscales; and parenting daily hassles, which consisted of behaviors and chores subscales. The results from the CFA showed that the measurement model fit the data very well, *χ^2^*(9) = 37.71, *p* < 0.01, CFI = 0.96, TLI = 0.93, RMSEA = 0.09, 90% CI [0.06–0.13], SRMR = 0.08. The standardized item loadings ranged from 0.41 to 0.97 across latent variables, meaning that all loadings were acceptable. Once the measurement model fit the data well, we moved forward with the structural model [40].

### 3.2. Structural Model

**Figure 1:**
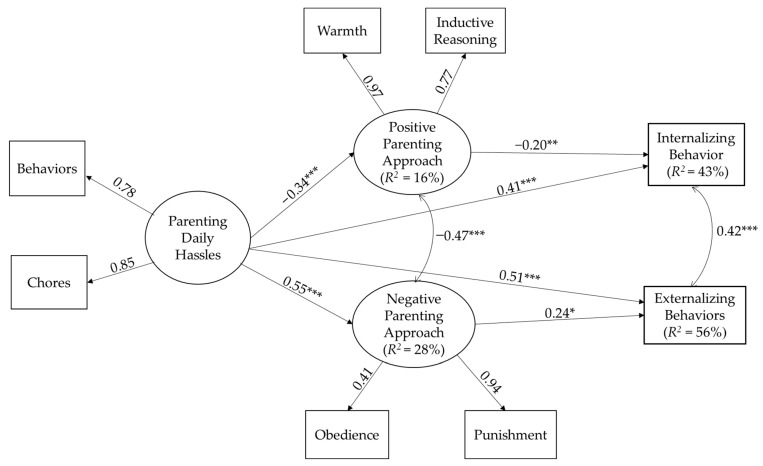
Parenting daily hassles predicting children’s internalizing and externalizing behaviors through positive and negative parenting approaches. *** *p* < 0.001, ** *p* < 0.01, * *p* < 0.05. Note. Only significant paths are depicted for brevity. Child sex (B = −0.13, *β* = −0.16, *p* < 0.01, favoring boys), child age (B = −0.006, *β* = −0.21, *p* < 0.001), family SES (B = −0.07, *β* = −0.15, *p* = 0.01), mother employment status (B = 0.15, *β* = 0.19, *p* < 0.01, favoring employed mothers) as a covariate was controlled for daily hassles. Child sex (B = 0.06, *β* = 0.11, *p* < 0.05, favoring girls) and child age (B = 0.002, *β* = 0.10, *p* < 0.05) as a covariate was controlled for the negative parenting approach. Fear of COVID-19 (B = 0.08, *β* = 0.19, *p* < 0.001) as a covariate was controlled for the negative parenting approach. Family SES (B = −0.82, *β* = −0.11, *p* < 0.05) and child age (B = −0.04, *β* = −0.10, *p* < 0.01) as a covariate was controlled for externalizing behaviors. Family SES (B = −0.97, *β* = −0.11, *p* < 0.05) and fear of COVID-19 (B = 1.27, *β* = 0.14, *p* < 0.01) as a covariate was controlled for internalizing behaviors.

As we followed a top-down model-building approach, in the first model we included all possible covariates, including child age, child sex, family SES, mother employment status during the pandemic, and fear of COVID-19 in the model. The results of SEM were as follows: *χ^2^*(30) = 139.294, *p* < 0.001, CFI = 0.91, RMSEA = 0.10, 90% CI [0.08, 0.12], SRMR = 0.07. As seen from the model fit indices, there was room for model improvement. In the competing nested model, we removed the nonsignificant paths from the covariates in the model. Results from the final structural model showed a better fit to the data: *χ^2^*(44) = 152.448, *p* < 0.001, CFI = 0.92, RMSEA = 0.08, 90% CI [0.71, 0.10], SRMR = 0.07. In the second model, the RMSEA value was better, indicating the model was improved [42]. In the parsimonious model, parenting daily hassle was positively related to children’s internalizing behaviors (*B* = 7.06 (*SE* = 1.15), *β* = 0.41, *p* < 0.001) and externalizing behaviors (*B* = 7.60 (*SE* = 1.08), *β* = 0.51, *p* < 0.001). Furthermore, the positive parenting approach was negatively related to children’s internalizing behaviors (*B* = −4.53 (*SE* = 1.78), *β* = −0.20, *p* < 0.01), and the negative parenting approach was positively related to children’s externalizing behaviors (*B* = 4.85 (*SE* = 2.05), *β* = 0.24, *p* < 0.05). The final model is depicted in Figure 1.

There was a significant indirect path from parenting daily hassles to children’s internalizing behaviors through the positive parenting approach (*β* = 0.07 (*SE* = 0.02), [95% CI: 0.01, 0.13]). In addition, there was an indirect path from parenting daily hassles to children’s externalizing behaviors through a negative parenting approach (*β* = 0.13 (*SE* = 0.05), [95% CI: 0.03, 0.25]). Nevertheless, testing mediating effect in the absence of longitudinal data would be problematic [45]; therefore, our significant result from this model could be interpreted as an indirect effect rather than a pure mediation. Overall, parents with higher levels of daily hassles showed lower positive parenting; in turn, their children showed higher levels of internalizing behaviors. In addition, parents with higher levels of daily hassles showed higher negative parenting; in turn, their children showed higher externalizing behaviors. See Supplementary Material (Figures S1 and S2) for graphical depictions of bootstrap distributions with bias-corrected 95% credible confidence intervals.

## 4. Discussion

We aimed to examine the contributions of parenting daily hassles and parenting approaches to child behavior problems during the COVID-19 pandemic with a particular interest in testing the indirect effect of daily hassles on children’s behavior problems via parenting approaches. We found a significant indirect effect of daily hassles on children’s internalizing behaviors via positive parenting. In addition, there was an indirect path from parenting daily hassles to children’s externalizing behaviors through the negative parenting approach. In the following sections, we discussed each result in turn.

### 4.1. Parenting Daily Hassles and Children’s Behavior Problems

Our first research question was to discover associations between parenting daily hassles and children’s behavior problems. In light of this question, we hypothesized that daily hassles would be positively associated with children’s behavior problems. The findings from the current study confirmed our expectations by showing that daily parenting hassles were positively related to children’s internalizing and externalizing behavior problems. In other words, parents’ experiences of day-to-day burdens stemming from caring for children contributed to children’s behavior problems. Consistent with our findings, previous studies also indicated that the daily hassles that emerge through parenting tasks undermine children’s behavior problems [22,23,24]. For instance, in a longitudinal study, Stone and her colleagues [46] found that parenting daily hassles when children were four years old were predictors of children’s internalizing and externalizing behavior problems for the next two subsequent years. Based on the previous studies and the current results, it may be suggested that parents with high levels of daily hassles could unintentionally or intentionally lead children to display higher levels of behavior problems.

### 4.2. Parenting Approaches and Children’s Behavior Problems

In another research question, we aimed to examine the association between parenting approaches and children’s behavior problems. We hypothesized that positive parenting approaches would lead children to display fewer behavior problems, and negative parenting approaches would lead them to exhibit higher levels of behavior problems. Results from the current study confirmed our hypothesis by revealing a statistically significant direct effect of parenting approaches on children’s behavior problems. Aligned with the present findings, previous studies also found that negative parenting approaches, such as punishment, were related to the frequency of children’s externalizing and internalizing behavior problems [23,45,47]. One possible explanation for why positive parenting practices (e.g., inductive reasoning and warmth) inhibit children from showing behavior problems, while negative parenting practices (e.g., punishment and obedience-demanding) could trigger children to display more frequent behavior problems may be that children model their parents in their behavior patterns [48]. Children exposed to negative parenting approaches may model their parents’ behavior and show it in different contexts, which may be the signs of externalizing behavior problems. Another possible explanation may be that using negative parenting approaches, such as demanding obedience and punishment, may hinder children from internalizing the parents’ messages, resulting in violation of rules and expressing externalizing behavior problems [49,50]. On the other hand, practicing positive parenting approaches has been consistently related to positive child outcomes [47]. Compared to negative parenting approaches, positive parenting practices, such as showing warmth and inductive reasoning, may help children understand the parents’ social messages and internalize their values, supporting children to practice positive behaviors and reducing their behavior problems.

Considering the cross-sectional nature of the current study, correlational results could be interpreted the other way around too, which is that children’s behavior problems may induce harsh parenting styles. Consistent with previous work [26,33], children who are temperamentally difficult (e.g., lack of self-regulation and higher reactivity) could lead parents to use negative parenting practices.

### 4.3. Indirect Effect of Parenting Daily Hassles on Children’s Behavior Problems through Parenting Approaches

Consistent with prior research, parents with high levels of daily hassles were likely to report higher levels of behavior problems in their children [22,24]. High parenting daily hassles may be seen as an environmental risk factor for children’s behavioral outcomes [23]. Although the evidence is clear that children of parents with high parenting daily hassles are likely to report frequent behavior problems in their children [22], the mechanism behind why daily hassles undermine children’s behavior problems can be explained more precisely by considering the parenting approach in the association between daily parenting hassles and children’s behavior problems.

In the current research, we examined the relationship between parenting daily hassles and children’s behavior problems through parenting approaches. Firstly, our findings showed the indirect effect of parenting daily hassles on children’s externalizing behaviors through negative parenting approaches. In detail, parents with more parenting daily hassles exhibited more negative parenting behaviors, which in turn tended to predict more externalizing behaviors in children. Thus, the association between parenting daily hassles and children’s externalizing behaviors grew to be accounted for by negative parenting approaches. This suggests that daily parenting hassles may have a worsening effect on externalizing behaviors through an association with negative parenting approaches [23].

Secondly, our findings showed the indirect effect of parenting daily hassles on children’s internalizing behaviors through positive parenting approaches. Parents with higher parenting daily hassles showed less positive parenting approaches, and in turn, their children showed higher levels of internalizing behaviors. This finding is congruent with previous studies, showing parenting daily hassles reflect stress and burden that could undermine positive parenting approaches [51,52]. In the current study, we found that when parents reported higher parenting daily hassles, they were more likely to report less positive parenting approaches, such as providing fewer explanations (inductive reasoning) and showing less warmth to their children, which naturally resulted in children displaying internalizing behavior problems. We should consider the fact that negative and positive parenting approaches in the same research model showed differential outcomes on children’s behavior problems. Based on the current study’s findings, we can suggest that positive and negative parenting approaches have differential roles in children’s behavior problems. These differential paths from parenting daily hassles to children’s behavioral problems are consistent with previous work with Turkish parents and children [23].

### 4.4. Practical Implications

Even though there are studies examining the relationship between parenting stress and child adjustment through an indirect path via parenting, the current study is unique to our knowledge in examining the pattern by focusing on parenting daily hassles, which include the daily parenting stress of parents as well as other minor stressors in day-to-day living. Our findings suggest that interventions focusing on stress management may be effective in reducing daily parenting hassles, which may lead to a decrease in practicing negative parenting strategies and, in turn, lead to a reduction in children’s behavior problems.

Given the indirect relationship between parenting daily hassles and children’s externalizing behavior problems, we can propose that in intervention programs aiming to reduce children’s externalizing behavior problems, it may be more effective to study parenting daily hassles and parents’ negative parenting approaches, such as punishment and obedience-demanding practices. On the other hand, in interventions aiming to reduce children’s internalizing behavior problems focusing on parenting daily hassles and positive parenting approaches may be more effective.

Furthermore, the current study provided evidence addressing the indirect effect of parenting daily hassles on children’s behavior problems through parenting approaches during the COVID-19 outbreak. During the COVID-19 lockdown, some parents may have faced an increased amount of parenting daily hassles compared to before the pandemic [53]. In order to overcome the adverse effects of the COVID-19 pandemic on parents, intervention studies may focus on reducing the parenting daily hassles in their day-to-day interactions with children. In addition, studying the family interactions and both parents’ support for each other in their role as parents might be explored in this model as it can reduce the perception of parenting daily hassles. Recent studies have shown that a spouse’s support in child-rearing issues during the COVID-19 was related to adaptive parenting behaviors [53]. Therefore, parents’ interaction during the COVID-19 might be crucial as it may be related to parenting daily hassles and, in turn to parents’ parenting approaches.

## 5. Study Limitations

There were some limitations to the present study. First, the nature of this study was cross-sectional, which limits making causal inferences from the results. Second, we solely relied on one mother’s reports for children’s behavior problems. In addition to maternal reports, direct observations and taking multi-informant reports may be more reliable in measuring children’s behavior problems [53]. Third, the present research only considered the maternal parenting approaches and parenting daily hassles in studying children’s behavior problems. There are studies showing the relationship between paternal parenting styles and children’s behavior problems [54]. Studying paternal parenting daily hassles in this model might be a unique contribution to the literature. Comparing the fathers’ perception of parenting daily hassles to that of the mothers and examining how it may affect their parenting behavior and, in turn, the children’s behavior problems, might be more explanatory in understanding the mediation mechanism. Fourth, including parents’ mental health in the study could provide a comprehensive picture of the baseline of parents’ and children’s behavioral outcomes.

## 6. Conclusions

In the current study, we examined the contributions of parenting daily hassles and parenting approaches to child behavior problems during the COVID-19 pandemic with a particular interest in testing the indirect effect of daily hassles on children’s behavior problems via parenting approaches. Daily hassles of the parents contributed to both their parenting and children’s behavior problems. Positive parenting practices negatively predicted internalizing behaviors, and negative parenting practices positively predicted externalizing behaviors. Finally, the daily hassles of parents undermined their positive parenting practices, which in turn negatively predicted children’s internalizing behaviors. In addition, the daily hassles of parents exacerbated negative parenting practices, which in turn increased children’s externalizing behaviors.

## Figures and Tables

**Table 1:** Bivariate correlations and descriptive statistics for the study variables.

Variables.	1	2	3	4	5	6	7	8	9	10	11	12	13
Externalizing Beh	-												
2. Internalizing Beh	0.64 **	-											
3. Warmth	−0.36 **	−0.39 **	-										
4. Inductive Reasoning	−0.38 **	−0.35 **	0.75 **	-									
5. Obedience	0.11 *	0.24 **	−0.12 *	−0.10	-								
6. Punishment	0.51 **	0.45 **	−0.49 **	−0.43 **	0.34 **	-							
7. DH_Behaviors	0.63 **	0.51 **	−0.32 **	−0.27 **	0.15 **	0.48 **	-						
8. DH_Chores	0.54 **	0.47 **	−0.24 **	−0.21 **	0.04	0.34 **	0.68 **	-					
9. COVID Fear	−0.03	0.09	0.18 **	0.14 **	0.31 **	0.01	−0.02	0.02	-				
10. Child Age	0.21 **	−0.04	0.02	0.14 **	0.05	−0.01	−0.15 **	−0.23 **	0.03	-			
11. Mother Age	−0.21 **	−0.10	0.07	0.17 **	0.02	−0.07	−0.18 **	−0.15 **	0.09	0.54 **	-		
12. Father Age	−0.19 **	−0.09	0.06	0.15 **	0.01	−0.09	−0.16 **	−0.13 *	0.09	0.45 **	0.81 **	-	
13. Family SES	−0.19 **	−0.18 *	0.01	0.15 **	−0.01	−0.01	−0.08	−0.15 **	−0.04	0.06	0.26 **	0.11 *	-
14. Child Sex	−0.12 *	−0.04	0.01	−0.01	0.03	0.04	−0.08	−0.18 **	0.01	0.02	0.01	−0.02	0.04
n	338	338	338	338	338	338	338	338	338	338	338	338	338
Mean	9.59	11.13	4.76	4.64	2.54	1.43	2.05	1.97	2.59	56.33	35.01	37.64	0.00
SD	6.07	7.09	0.32	0.42	0.73	0.32	0.55	0.45	0.78	15.14	4.14	4.46	0.82
Range	0–31	0–39	3.67–5	3.14–5	1–4.83	1–2.75	1–3.86	1–3.33	1–4.71	21–89	24–47	28–50	−3.20–2.23
Skewness	0.882	0.795	−1.428	−1.123	0.200	1.218	0.392	0.018	0.068	−0.106	−0.038	0.159	−1.252
Kurtosis	0.659	0.304	1.333	0.405	−0.180	2.057	0.018	−0.323	-0.327	−0.979	−0.345	−0.402	2.792

Note. * *p* < 0.05, two-tailed. ** *p* < 0.01, two tailed. Sex: 1 = Girl, 0 = Boy. Beh: Behaviors. DH = Daily Hassles.

## Data Availability

The datasets generated during and/or analyzed during the current study are available from the corresponding author upon reasonable request.

## Supplementary Materials

The following supporting information can be downloaded at: https://www.mdpi.com/article/10.3390/children10020312/s1, Bootstrap distributions of indirect effects; Figure S1: Parenting daily hassles to internalizing behaviors via positive parenting approach; Figure S2: Parenting daily hassles to eternizing behaviors via negative parenting approach.
