# Supplementary material for: Examining the Contributions of Parents’ Daily Hassles and Parenting Approaches to Children’s Behavior Problems during the COVID-19 Pandemic
Source: Children (Basel). 2023 Feb 7;10(2):312. doi: 10.3390/children10020312 (PMC10004077; doi:10.3390/children10020312)
Supplement: Supplementary file 1 [file children-10-00312-s001.zip › children-2191238-supplementary.pdf]

Supplementary Materials Bootstrap distributions of indirect effects

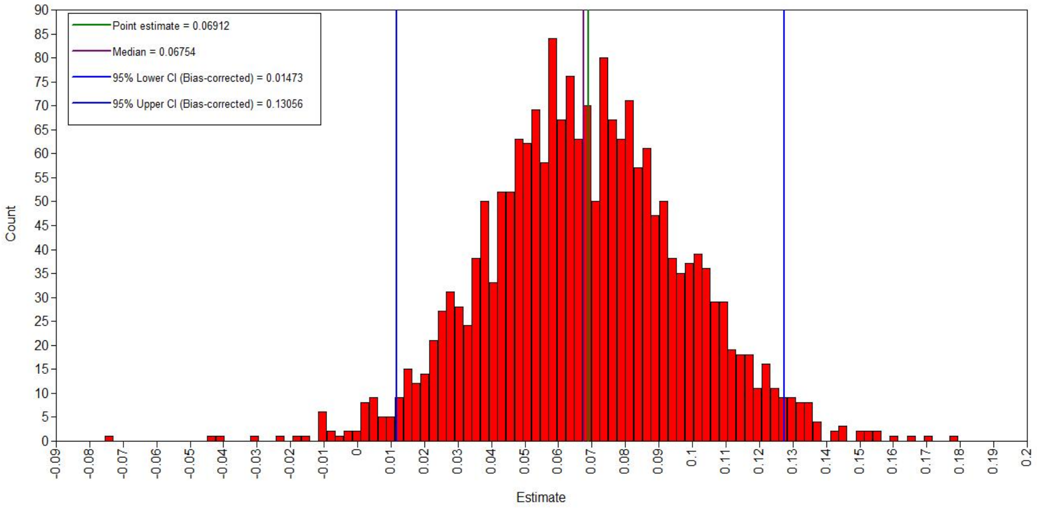

Figure S1. Parenting daily hassles to internalizing behaviors via positive parenting approach.

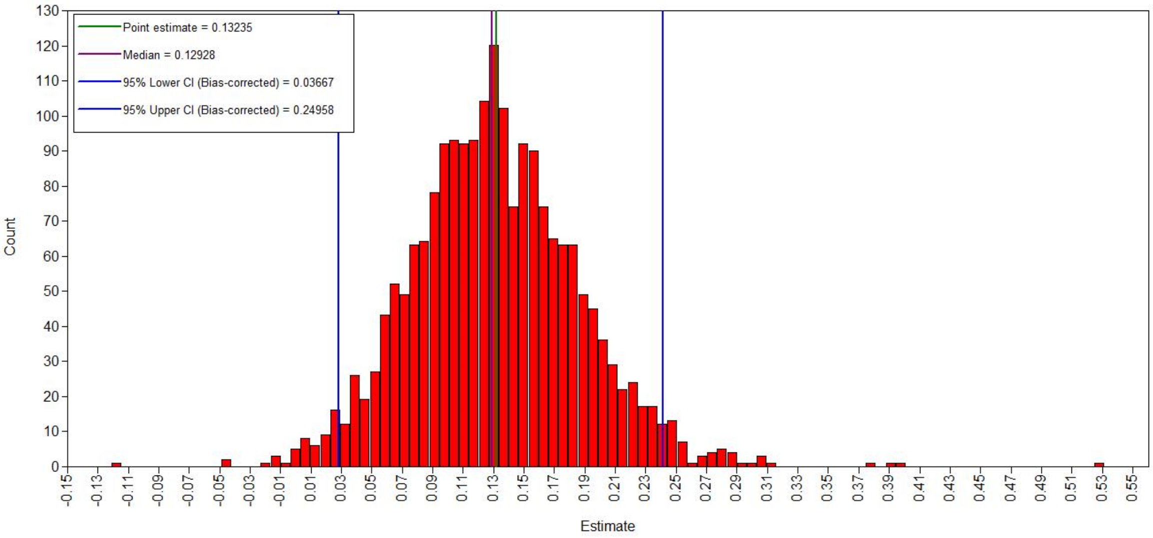

Figure S2. Parenting daily hassles to eternizing behaviors via negative parenting approach.
